# Supplementary figures and images for: APOBEC3A-Induced DNA Damage Drives Polymerase θ Dependency and Synthetic Lethality in Cancer
Source: bioRxiv. 2025 Oct 22:2025.10.21.682588. Preprint. [Version 1] doi: 10.1101/2025.10.21.682588 (PMC12633405; doi:10.1101/2025.10.21.682588)

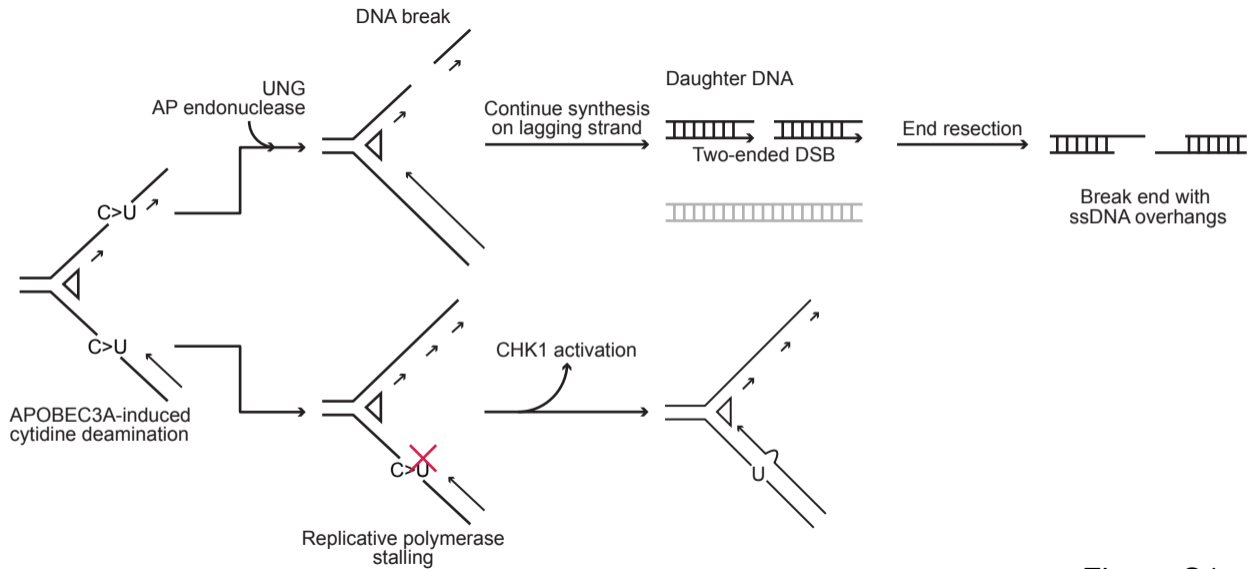

Figure S1

Supplement: Supplement 1 — Supplementary Figure S1. Schematic model for the formation of DSB induced by APOBEC3A. Breaks occur in the template strand while lagging-strand synthesis resumes in the APOBEC3A-loaded replication fork, leaving intact Okazaki fragments flanking the break, generating daughter DNA with two-ended DSBs. [file media-1.pdf]

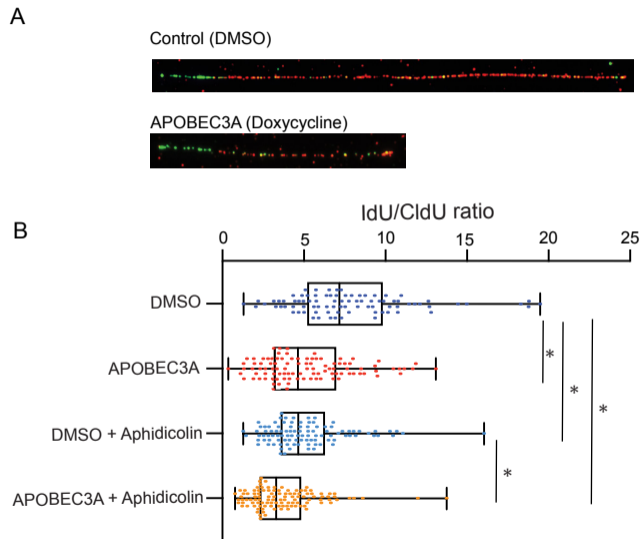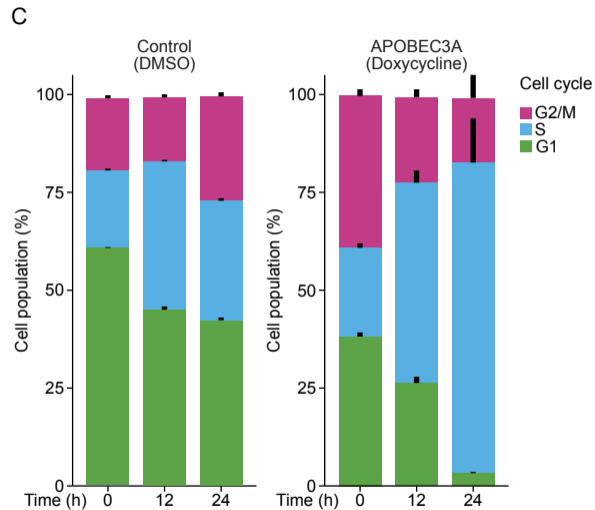

Figure S2

Supplement: Supplement 2 — Supplementary Figure S2. APOBEC3A expression causes replication-fork stalling and cell cycle arrest in S phase. A, Representative DNA fiber images from cells exposed to APOBEC3A or DMSO control, showing altered replication dynamics. B, Quantification of the IdU to CldU length ratio in 5637 cells illustrates a significant reduction in the length of nascent DNA fragments in APOBEC3A-expressing cells compared to DMSO control. Aphidicolin-treated cells serve as control for replication inhibition. 80–100 fibers per condition were quantified from two independent experiments. A Box plot with individual data points shows median with interquartile range, and the whiskers span from minimum to maximum value. One-way ANOVA with multiple comparisons. *: p < 0.05. C, Bar chart illustrating the distribution of cells across different cell cycle stages showing enrichment in S-phase cells induced by APOBEC3A. [file media-2.pdf]

A

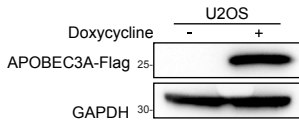

B

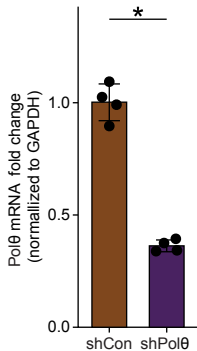

Figure S3

Supplement: Supplement 3 — Supplementary Figure S3. APOBEC3A induction in the U2OS cell line and shRNA mediated Polθ inhibition. A, SDS-PAGE western blotting showing the inducible expression levels of APOBEC3A-Flag in U2OS cells. B, Efficiency of Polθ knockdown (2.5 fold) using shRNA in 5637 cells with doxycycline-inducible APOBEC3A, measured by quantitative PCR. Each dot represented one replicate. Two-sided t-test. Data represent mean ± SD [file media-3.pdf]

scramble

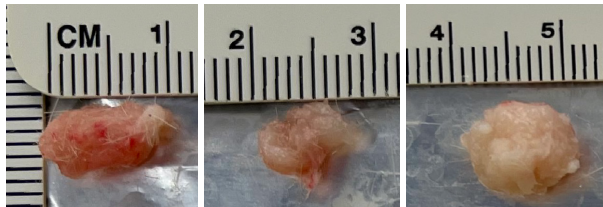

shPolθ

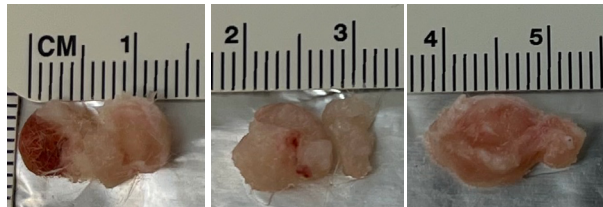

scramble + APOBEC3A<sup>OE</sup>

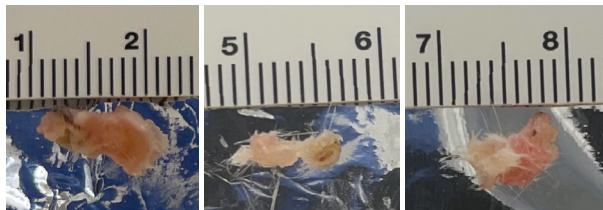

shPolθ + APOBEC3A<sup>OE</sup>

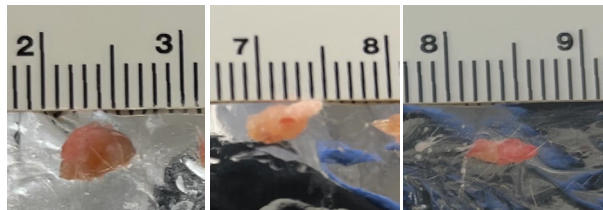

Figure S4

Supplement: Supplement 4 — Supplementary Figure S4. Representative resected macroscopic tumor images from scramble and shPolθ xenografts with and without APOBEC3A. Representative images of 3 resected tumors from each experimental group (scrambled, shPolθ, scramble+APOBEC3AOE, and shPolθ +APOBEC3AOE at study endpoint (day 56). shPolθ +APOBEC3AOE shows the smallest size among groups. Scale placed beside tumor during taking picture (millimeters) for reference. [file media-4.pdf]
